# Risk factors for mortality among Tanzanian infants and children

**DOI:** 10.1186/s41182-020-00233-8

**Published:** 2020-06-04

**Authors:** Rodrick R. Kisenge, Chris A. Rees, Jacqueline M. Lauer, Enju Liu, Wafaie W. Fawzi, Karim P. Manji, Christopher P. Duggan

**Affiliations:** 1 Department of Paediatrics and Child Health, Muhimbili University of Health and Allied Sciences, P.O. Box 65001, Dar es Salaam, Tanzania; 2 Division of Emergency Medicine, Boston Children’s Hospital, Harvard Medical School, Boston, MA USA; 3 Clinical Research Centre, Division of Gastroenterology, Hepatology and Nutrition, Boston Children’s Hospital, Boston, MA USA; 4 Institutional Centers for Clinical and Translational Research, Boston Children’s Hospital, Boston, MA USA; 5 Department of Global Health and Population, Harvard School of Public Health, Boston, MA USA; 6 Division of Gastroenterology, Hepatology and Nutrition, Boston Children’s Hospital, Boston, MA USA; 7 Center for Nutrition, Division of Gastroenterology, Hepatology, and Nutrition, Boston Children’s Hospital, Boston, MA USA

**Keywords:** Mortality, Pediatric, Infants, HIV, Tanzania

## Abstract

**Background:**

During the era of the Millennium Development Goals, under 5 mortality rates decreased significantly worldwide; however, reductions were not equally distributed. Children in sub-Saharan Africa still account for more than 50% of the world’s annual childhood deaths among children under 5 years of age. Understanding upstream risk factors for mortality among children may reduce the large burden of childhood mortality in sub-Saharan Africa. Our objective was to identify risk factors for mortality among infants and children in Tanzania.

**Methods:**

We conducted a secondary analysis of data pooled from two randomized-controlled micronutrient supplementation trials. A total of 4787 infants were enrolled in the two trials (*n* = 2387 HIV-exposed and *n* = 2400 HIV-unexposed). Predictors of mortality were assessed using unadjusted and adjusted hazard ratios (aHRs).

**Results:**

There were 307 total deaths, 262 (11%) among children who were HIV-exposed and 45 (2%) among children who were HIV-unexposed (*P* < 0.001). The most common cause of death was respiratory diseases (*n* = 109, 35.5%). Causes of death did not significantly differ between HIV-exposed and HIV-unexposed children. In adjusted regression analyses, children with birth weight <2500 g (aHR 1.75, 95% CI 1.21–2.54), Apgar score of ≤7 at 5 min (aHR 2.16, 95% CI 1.29–3.62), or who were HIV-exposed but not infected (aHR 3.35, 95% CI 2.12–5.28) or HIV-infected (aHR 27.56, 95% CI 17.43–43.58) had greater risk of mortality.

**Conclusions:**

Infection with HIV, low birthweight, or low Apgar scores were associated with higher mortality risk. Early identification and modification of determinants of mortality among infants and children may be the first step to reducing such deaths.

## Background

During the era of the Millennium Development Goals, the under 5 mortality rate decreased by 53% worldwide [1]; however, reductions were not equally distributed. Children in sub-Saharan Africa still account for more than 50% of the world’s annual childhood deaths among children under 5 years of age [2]. Many factors contribute to high rates of childhood mortality in sub-Saharan Africa, including high prevalence of human immunodeficiency virus (HIV), limited access to skilled healthcare providers, long distances to healthcare facilities, and caregiver delays in care-seeking for their children [3–6].

The implementation of triage systems has been shown to decrease inpatient mortality among children in sub-Saharan Africa [7, 8] and early recognition scores are being developed to identify patients at risk of inpatient mortality [9–11]. However, relatively more work describing mortality in children in sub-Saharan Africa has focused on the inpatient setting, largely neglecting periods before and after hospitalization [12].

Tanzania, a populated country in East Africa, has made tremendous strides in decreasing under 5 mortality. The under 5 mortality rate is now 53/1000 live births, a threefold decline over the past 30 years [13]. However, the drop in under 5 mortality has primarily been due to decreases in child mortality rates [14], leaving infants and young children susceptible to early death. Further understanding mortality among young children is the first step towards further reducing mortality among Tanzanian children.

Understanding upstream risk factors for mortality among children prior to hospital admission and in the outpatient setting could reduce the large burden of childhood mortality in sub-Saharan Africa. The objective of this study was to identify maternal and child risk factors for mortality among children enrolled in two, large, randomized controlled trials in Dar es Salaam, Tanzania. We hypothesized that children who died in these trials would have different demographic, socioeconomic, and clinical factors than those who survived.

## Methods

### Study design

We conducted a secondary analysis of two, randomized-controlled micronutrient supplementation trials conducted in Dar es Salaam, Tanzania (NCT00197730 and NCT00421668). These trials were conducted from 2004 to 2007 and 2007 to 2009, respectively. Details of the methodology and main results for both trials have previously been reported [15, 16]. Briefly, in the first trial, consenting pregnant women ≥18 years presenting at ≤32 weeks gestation were tested for HIV at antenatal clinics. Mothers who tested positive for HIV were enrolled and, at 6 weeks of age, their infants were randomized to receive either a daily oral dose of multivitamins or placebo for 24 months. In the second 2 × 2 factorial design trial, women were similarly screened for HIV at ≤32 weeks gestation, but only HIV-negative women were enrolled. The mean duration of follow-up was similar in the two trials (18.1 months [standard deviation ±7.5] among children born to HIV-positive mothers and 18.8 months [standard deviation ±4.2] among children born to HIV-negative mothers. Apgar scores were assigned by birth attendants and recorded in each infant’s newborn form. Apgar scores were transcribed by study staff at the time of enrolment. At 6 weeks of age, infants were randomized to receive either a daily oral dose of multivitamins, zinc, multivitamins + zinc, or placebo for 18 months.

Both trials included participants who were singleton births born to women ≥18 years of age who presented for prenatal care at the 32nd week of gestation or earlier. Both trials excluded infants who were born of multiple gestation, had serious congenital anomalies, and those whose mothers chose not to participate in the micronutrient trial. In both trials, participants were asked to provide personal and household information meant to inform the socioeconomic status of the household. Variables to establish socioeconomic status included maternal employment status, amount spent on food per person, and household assets including possession of sofa, radio, fan, television or refrigerator. Subsequently, household wealth index was created in each trial through a principal component analysis of the household assets.

In both trials, monthly follow-up clinic visits were performed to assess infant and child morbidity based on caregivers’ description of symptoms present during the previous month. To minimize recall bias, caregivers kept symptom diaries at home in which they recorded symptoms and care sought during each month. These diaries included pictures that aided caregivers in identifying symptoms (e.g., cough, vomiting, diarrhea, etc.). Caregivers were asked to check off days the child had corresponding symptoms. During the monthly clinic visits, caregivers presented the symptom diaries to study staff for review. During the monthly clinic visits, trained nurses assessed anthropometric measurements using standardized techniques [17]. Caregivers reported monthly estimates of the amount they spent for food for each person in the household recorded in Tanzanian shillings during the study period. Children who missed scheduled follow-up appointments were visited at home by study nurses to minimize loss to follow-up.

All mothers and children who were HIV-exposed received medical care according to Tanzanian guidelines for the care of HIV-infected individuals at the time these trials were conducted [18, 19]. In accordance with the Tanzanian national HIV guidelines at the time, mothers with HIV were eligible for antiretroviral therapy if they had a World Health Organization (WHO) stage IV disease, CD4 counts ≤200 cells/mL, or WHO stage III disease and CD4 cell counts of ≤350 cells/mL. Children were eligible for antiretroviral therapy if their CD4 percentage was <20% or if they had pediatric WHO stage III or IV disease [18, 19]. In keeping with contemporary recommendations, mothers who were infected with HIV also received supplements which contained high-dose vitamins B complex, vitamin C, and vitamin D during pregnancy and through lactation. Prevention of mother to child transmission therapy at the time was limited to nevirapine prophylaxis given in two doses. A single dose of nevirapine was given to the mother at the time of the onset of labor and a second dose to the infant within 72 h of birth. Infants born to HIV-infected mothers also received prophylactic trimethoprim and sulfamethoxazole for 6 months and as long as the child is breastfed. HIV testing was done for all infants in the first trial at 6 weeks of age using the Amplicor HIV-1 DNA assay (version 1.5, Roche Molecular Systems, Inc., Pleasanton, California, USA). Children in the first trial were tested for HIV again at 18 months of age using HIV ELISA assay followed by Enzygnost anti-HIV-1/2 Plus (Dade Behring, Deerfield, Illinois, USA). All children in both trials received immunizations as per Tanzanian vaccination schedules, vitamin A supplements, and medical care for all illnesses.

The standard definitions of diseases diagnosed among children during the study period were as described in the WHO definitions and guidelines [20]. In case of child death, a verbal autopsy was performed in the home of the family to determine the cause of death. Verbal autopsy forms were coded independently by two pediatricians, and any differences were resolved by a third pediatrician.

All caregivers provided written informed consent for enrolment in the trials. Ethical approval for both trials was granted by the Muhimbili University of Health and Allied Science Committee of Research and Publications, the Tanzanian National Institute of Medical Research, the Tanzanian Food and Drug Authority, and the Harvard T.H. Chan School of Public Health Human Subjects Committee.

### Statistical analysis

Descriptive statistics were calculated to evaluate baseline characteristics among infants in the two trials. Mean (standard deviation) was presented for continuous variables and *n* (%) for categorical variables. A household wealth index was created in each trial through a principal component analysis of household assets, including possession of sofa, radio, fan, TV, and refrigerator. Cox proportional hazards models were used to estimate hazard ratios (HRs) and corresponding 95% confidence intervals (CIs) for time to death. Variables in univariate analysis with *P* < 0.10 were included in multivariate analysis model. Finally, a Kaplan-Meier survival curve to compare cumulative mortality between HIV-infected, HIV-exposed but uninfected, and HIV-unexposed children was created. Analyses were performed using SAS Version 9.4 (SAS Institute, Cary, NC, USA). *P* values <0.05 were considered statistically significant.

## Results

A total of 4787 infants were enrolled in the two trials (2,387 infants were HIV-exposed and 2400 were HIV-unexposed). There were 2505 (52.3%) males enrolled and 647 (14.2%) of enrolled participants were born prematurely. There were 307 deaths, 262 among children born to HIV-infected mothers and 45 among children born to HIV-negative mothers. The vast majority (95.4%, *n* = 293) of deaths in both trials occurred in the first 18 months and the distribution of deaths over 18 months did not differ between the two trials (*P* = 0.41). The mean age at the time of death was 7.3 months (standard deviation ±5.2) among children born to HIV-infected mothers and 9.2 months (standard deviation ±5.1) among children born to HIV-negative mothers. The mortality rate among children born to HIV-infected mothers (11%) was substantially higher compared to children born to HIV-negative mothers (2%) (*P* < 0.001). Maternal, socioeconomic, and infant characteristics are described in Table 1. Mothers of enrolled children had a mean age of 27.3 years at the time of enrolment (SD ± 5.1) and most mothers had 1 to 7 years of formal education (*n* = 3,431, 72.2%). A majority of mothers reported spending <1000 Tanzanian shillings per person per day on food (*n* = 2755, 60.8%). The mean duration of exclusive breastfeeding was 2.7 months (SD ± 2.0) and the mean duration of any breastfeeding was 8.8 months (SD ± 6.3).

**Table 1:** Maternal, socioeconomic, and infant characteristics of two cohorts in Dar es Salaam, Tanzania

	Total (***n*** = 4787)	Children of HIV-positive mothers (***n*** = 2387)	Children of HIV-negative mothers (***n*** = 2400)
Maternal characteristics			
Age in years (mean ± SD)	27.3 ± 5.1	28.2 ± 4.9	26.4 ± 5.0
Height in centimeters (mean ± SD)	155.8 ± 6.1	155.2 ± 6.0	156.5 ± 6.2
Weight in kilograms (mean ± SD)	60.6 ± 11.4	59.0 ± 10.9	62.2 ± 11.7
Middle upper arm circumference in centimeters (mean ± SD)	26.4 ± 3.2	25.9 ± 3.2	27.0 ± 3.1
Married or cohabitating with partner			
Yes	4206 (88.7%)	2051 (86.9%)	2155 (90.6%)
No	534 (11.3%)	310 (13.1)	224 (9.4)
Prior pregnancies (mean ± SD)	1.4 ± 1.3	1.5 ± 1.3	1.3 ± 1.4
Socioeconomic characteristics			
Formal education in years			
None	194 (4.1%)	158 (6.7%)	36 (1.5%)
1–7	3431 (72.2%)	1700 (71.9%)	1731 (72.5%)
≥8	1128 (23.7%)	507 (21.4%)	621 (26.0%)
Employment			
Housewife with income	550 (22.9%)	200 (8.7%)	350 (14.7%)
Housewife without income	2973 (62.1%)	1528 (66.5%)	1445 (60.8%)
Other^1^	1151 (12.7%)	570 (24.8%)	581 (24.5%)
Per person daily food expenditure <1000 Tanzanian shillings			
Yes	2755 (60.8%)	2101 (93.7%)	654 (28.6%)
No	1774 (39.2%)	141 (6.3%)	1633 (71.4%)
Household wealth index >75 percentile^2^			
Yes	1175 (24.8%)	577 (24.4)	598 (25.1%)
No	3569 (75.2)	1784 (75.6%)	1785 (74.9%)
Infant and child characteristics			
Infant sex			
Male	2505 (52.3%)	1289 (54.0%)	1216 (50.6%)
Female	2282 (47.7%)	1098 (46.0%)	1184 (49.3%)
Preterm, <37 weeks gestation			
Yes	647 (14.2%)	357 (15.2%)	290 (12.2%)
No	3905 (85.8%)	1995 (84.8%)	1910 (86.8%)
Low birth weight, <2500 g			
Yes	243 (5.2%)	161 (7.0%)	82 (3.5%)
No	4421 (94.8%)	2128 (93.0%)	2293 (96.5%)
Apgar ≤7 at 5 min after birth			
Yes	128 (2.9%)	87 (4.1%)	41 (1.9%)
No	4220 (97.1%)	2049 (95.9%)	2171 (98.1%)
HIV status			
HIV-infected	355 (7.5%)	355 (15.0%)	0 (0.0%)
HIV-exposed but uninfected	2005 (42.1%)	2005 (85.0%)	0 (0.0%)
HIV-unexposed	2400 (50.4%)	0 (0.0%)	2400 (100%)
Any breastfeeding in months (mean ± SD)	8.8 ± 6.3	4.4 ± 2.5	13.2 ± 5.9
Exclusive breastfeeding in months (mean ± SD)	2.7 ± 2.0	3.5 ± 2.1	1.88 ± 1.54
Exclusive breastfeeding in month categories			
None	1044 (22.0)	329 (13.8)	715 (30.2)
<3 months	1850 (39.0)	663 (27.9)	1187 (50.2)
≥3 months	1850 (39.0)	1387 (58.3)	463 (19.6)

^1^Other employment included businesswomen, work at a public house or restaurant, professional employment including being a teacher, nurse, etc., skilled office work, and unskilled employment

^2^Household wealth index was created separately for each study

The most common cause of mortality in both cohorts was respiratory diseases (Table 2). Cause of death could not be established in 26 children because the caregivers were unavailable for verbal autopsy. There were no statistically significant differences between the causes of deaths among HIV-infected infants and children and those who were not exposed to HIV.

**Table 2:** Primary causes of child death among 307 children in Dar es Salaam, Tanzania

	Total, ***n*** (%)	Children born to HIV positive mothers (***N*** = 262), ***n*** (%)	Children born to HIV negative mothers (***N*** = 45), ***n*** (%)
Respiratory diseases^1^	109 (35.5)	96 (36.6)	13 (28.9)
Malaria	53 (17.3)	46 (17.6)	7 (15.6)
Diarrheal diseases^2^	39 (12.7)	34 (13.0)	5 (11.1)
Undetermined	26 (8.5)	19 (7.3)	7 (15.6)
Meningitis	22 (7.2)	18 (6.9)	4 (8.8)
Septicaemia	20 (6.5)	20 (7.6)	0 (0.0)
Viral encephalitis	17 (5.5)	14 (5.3)	3 (6.6)
Other^3^	13 (4.2)	9 (3.4)	3 (6.7)
Neonatal conditions^4^	8 (2.6)	6 (2.3)	2 (4.4)
Total deaths	307 (100)	262 (100)	45 (100)

^1^Respiratory illnesses included upper respiratory infection, acute pneumonia and pulmonary tuberculosis

^2^Diarrheal diseases included acute watery diarrhea, persistent diarrhea and dysentery

^3^Other included burns, protein energy malnutrition, severe anemia in congestive cardiac failure, motor traffic accident and burns

^4^Neonatal conditions included sudden infant death syndrome, congenital anomalies and haemorrhagic disease of the newborn

In unadjusted analyses, several maternal, socioeconomic, and child-level factors were associated with mortality (Table 3). Children whose mothers were between 24 and 29 years old (HR 1.61, 95% CI 1.17–2.20, *P* < 0.003) and ≥ 30 years old (HR 1.43, 95% CI 1.03–2.00, *P* < 0.03) were at greater mortality risk compared to children whose mothers were below 24 years old. Children of mothers with a MUAC of <23 cm also had higher risk of mortality compared to children of mothers who had a MUAC of ≥23 cm (HR 1.58, 95% CI 1.15–2.17, *P* = 0.005). Children whose mothers had an income had reduced risk of mortality compared to mothers who did not have an income (HR 0.56, 95% CI 0.36–0.87, *P* < 0.001). Children whose mothers had a household wealth index of >75 percentile had lower risk of mortality when compared to children of mothers who had a household wealth index of ≤75 percentile (HR 0.73, 95% CI 0.55–0.97, *P* < 0.003). Children living in households in which <1000 Tanzanian shillings were spent per person per day for food had higher risk of mortality when compared to households in which ≥1000 Tanzanian shillings were spent on food per person each day (HR 3.34, 95% CI 2.45–4.56, *P* < 0.001). Infants born before 37 weeks gestation had elevated risk of mortality in comparison to infants born after 37 weeks gestation (HR 1.44, 95% CI 1.08–1.92, *P* = 0.01) and those with birthweight <2500 g also had higher risk of mortality when compared to infants with birthweight ≥2500 g (HR 2.91, 95% CI 2.05–4.12, *P* < 0.001). Infants who had an Apgar score ≤ 7 at 5 min were at higher risk of mortality compared to those with Apgar scores between 8 and 10 at 5 min (HR 2.14, 95% CI 1.29–3.54, *P* = 0.003). Infants and children who were HIV-exposed but uninfected (HR 3.30, 95% CI 2.35–4.68, *P* < 0.001) and infected with HIV (HR 26.56, 95% CI 18.93–37.28, *P* < 0.001) were also at greater mortality risk compared to children who were not exposed to HIV.

**Table 3:** Risk factors for mortality among infants and children in Dar es Salaam, Tanzania

	***n***/***N*** (%)	Unadjusted hazard ratio (95% CI)	***P*** value	Adjusted hazard ratio (95% CI)	***P*** value
Maternal characteristics					
Age (years)					
<24	53/1167 (4.5%)	Referent		Referent	
24–29	145/1986 (7.3%)	**1.61 (1.17–2.20)**	**0.003**	1.03 (0.74–1.43)	0.88
≥30	99/1518 (6.5%)	**1.43 (1.03–2.00)**	**0.03**	0.83 (0.58–1.18)	0.30
Middle upper arm circumference in centimeters					
≥23	258/4196 (6.2%)	Referent		Referent	
<23	45/478 (9.4%)	**1.58 (1.15–2.17)**	**0.005**	0.98 (0.71–1.35)	0.90
Married or cohabitating with partner					
Yes	257/4173 (6.2%)	0.74 (0.53–1.02)	0.06	0.95 (0.67–1.35)	0.78
No	43/527 (8.2%)	Referent		Referent	
Prior pregnancies					
0	67/1268 (5.3%)	Referent			
1–4	222/3307 (6.7%)	1.25 (0.95–1.64)	0.11		
≥5	11/133 (8.3%)	1.57 (0.83–2.97)	0.17		
Socioeconomic characteristics					
Formal education in years					
≥8	52/1120 (4.6%)	0.65 (0.36–1.16)	0.15		
1–7	235/3.400 (6.9%)	0.97 (0.56–165)	0.90		
None	14/193 (7.3%)	Referent			
Employment					
Housewife without income	208/2,950 (7.1%)	Referent		Referent	
Housewife with income	22/545 (4.0%)	**0.56 (0.36–0.87)**	**0.001**	0.70 (0.45–1.09)	0.11
Other	67/1,139 (5.9%)	0.84 (0.64–1.10)	0.21	0.95 (0.71–1.27)	0.72
Household wealth index >75 percentile					
No	241/3540 (6.8)	Referent		Referent	
Yes	58 (1106 (5.0)	0.73 (0.55–0.97)	0.003	0.77 (0.58–1.04_)	0.09
Per person daily food expenditure					
≥1000 Tanzanian shillings	48/1,749 (2.7%)	Referent			
<1000 Tanzanian shillings	238/2742 (8.7%)	**3.34 (2.45–4.56)**	**<0.001**	0.89 (0.59–1.35)	0.59
Infant characteristics					
Sex					
Female	136/2263 (6.0%)	Referent			
Male	171/2484 (6.9%)	1.15 (0.92–1.44)	0.22		
Preterm, <37 weeks gestation					
No	244/3,874 (6.3%)	Referent		Referent	
Yes	56/640 (8.8%)	**1.44 (1.08–1.92)**	**0.01**	1.16 (0.85–1.58)	0.35
Low birth weight, <2500 g					
No	246/4382 (5.6%)	Referent			
Yes	36/242 (14.9%)	**2.91 (2.05–4.12)**	**<0.001**	**1.74 (1.20–2.53)**	**0.004**
Apgar score at 5 min					
8–10	257/4182 (6.2%)	Referent		Referent	
≤7	16/128 (12.5%)	**2.14 (1.29–3.54)**	**0.003**	**2.08 (1.24–3.49)**	**0.006**
HIV status					
Unexposed	45/2360 (1.9%)	Referent		Referent	
Exposed but uninfected	119/2005 (5.9%)	**3.30 (2.35–4.68)**	**<0.001**	**3.95 (2.49–6.24)**	**<0.001**
Infected	133/355 (37.5%)	**26.56 (18.93–37.28)**	**<0.001**	**32.19 (20.26–51.16)**	**<0.001**
Exclusive breastfeeding duration					
Never	55/1034 (6.5%)	Referent			
<3 months	109/1832 (6.3%)	*1.14 (0.82–1.58)*	0.02	1.15 (0.82–1.61)	0.43
≥3 months	140/1840 (7.6%)	*1.43 (1.05,1.96)*	0.54	0.75 (0.54–1.05)	0.09

In adjusted analysis, infants and children with birth weight <2500 g had greater risk of mortality when compared to those with birthweight ≥2500 g (aHR 1.74, 95% CI 1.20–2.53, *P* = 0.004). Infants who had an Apgar score of ≤7 at 5 min had elevated risk of mortality compared to those who had Apgar scores of 8–10 (aHR 2.08, 95% CI 1.24–3.49, *P* = 0.006). The greatest risk of mortality was conferred by HIV status as infants and children who were HIV-exposed but not infected (aHR 3.95, 95% CI 2.49–6.24, *P* < 0.001) and those HIV-infected (aHR 32.19, 95% CI 20.26–51.16, *P* < 0.001) had greater risk of mortality when compared to children who were not exposed to HIV. Household wealth, maternal employment, and daily amount spent on food were not significantly associated with mortality in the multivariable analysis.

**Fig. 1:**
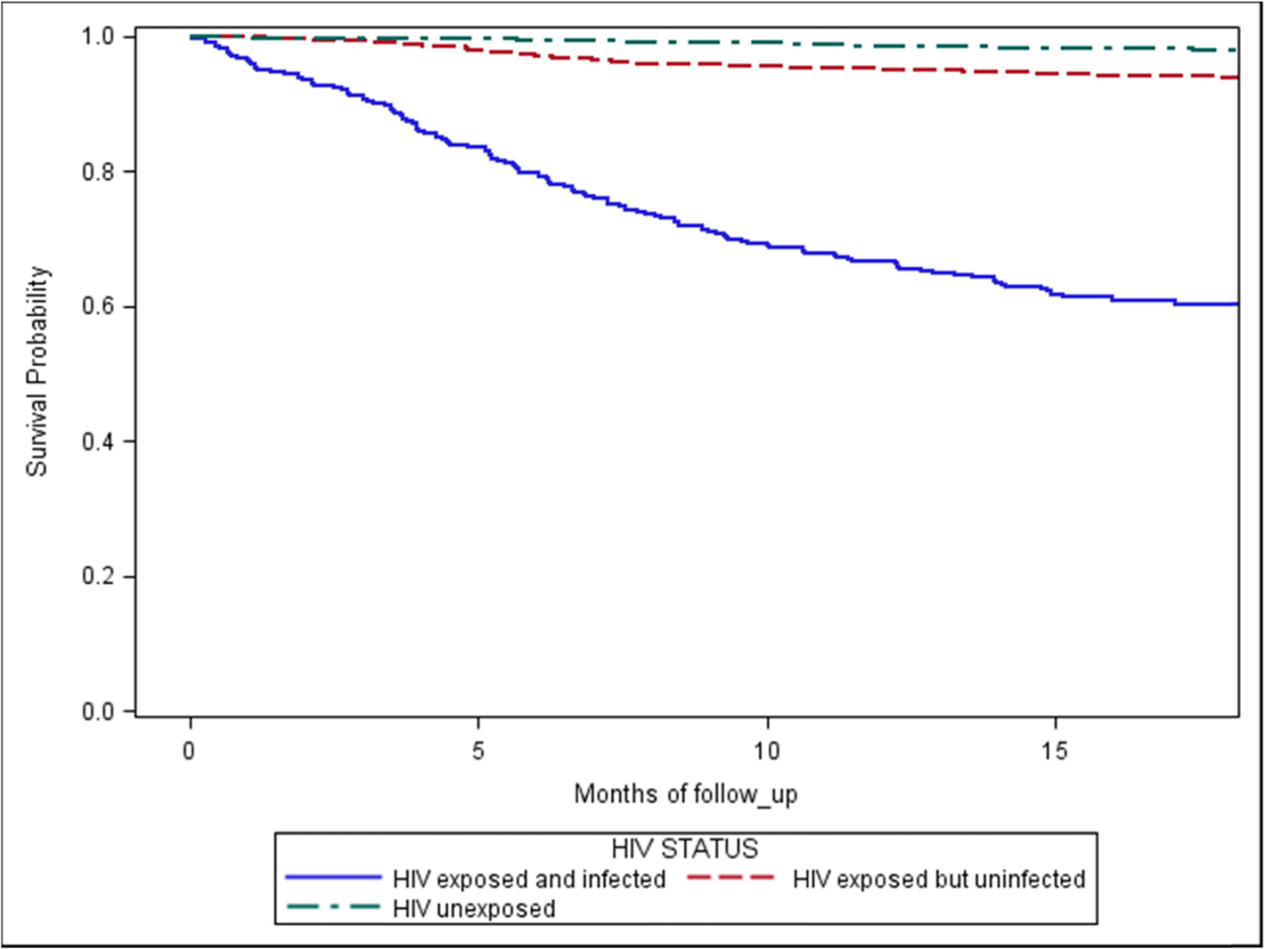
Survival curves of HIV-infected, HIV-exposed but uninfected, and HIV-unexposed children

The survival curve demonstrates that HIV-infected children had lower survival rates compared to those who were HIV-exposed but uninfected who, in turn, had lower survival compared to HIV-negative children (Fig. 1).

## Discussion

In this study with monthly follow-up over nearly 2 years, mortality risk among HIV-exposed infants and children was significantly higher than the mortality risk among infants and children who were not exposed to HIV. In multivariable analysis, low birthweight and lower Apgar scores were associated with greater infant and childhood mortality. Indicators of low socioeconomic status were not associated with increased risk of mortality among infants and children in this study. Despite marked disparities in mortality among HIV-infected infants and children and those who were not infected, the causes of death did not differ by HIV status. Presence of low Apgar scores and low birthweight at the time of delivery were associated with higher mortality risk among infants and children in our cohort.

Consistent with prior studies conducted in outpatient settings [21, 22], we found higher rates of mortality among HIV-infected infants and children compared to those who were HIV-negative. Previous work has demonstrated that child factors such as young age, malnutrition, and advanced WHO HIV staging may contribute to greater mortality among children with HIV compared to children who are not infected [23–25]. Though our secondary analysis did not include WHO HIV staging, our cohort similarly demonstrated malnutrition, manifesting as low birthweight, was associated with elevated mortality risk. Infants and children who are HIV-infected and are not started on antiretroviral therapy have higher mortality when compared to older children [23]. Mounting evidence on universal test and treat approaches for HIV reducing the incidence of HIV [26–28] has led to changes in country-level HIV guidelines. In 2013, the WHO recommended initiating antiretroviral therapy for all HIV-infected children under 5 years of age, regardless of CD4 cell count or WHO clinical stage [29]. This became policy in Tanzania in 2015 [30], which came after this study’s implementation. As our study results predate this policy change, there may have been elevated observed mortality rates among HIV-infected infants and children compared to contemporary mortality rates among HIV-infected infants and children as timely initiation of antiretroviral therapy reduces mortality among children living with HIV [23]. Nevertheless, the mortality rate observed among infants and children infected with HIV in this study may be similar to that in areas with low antiretroviral coverage, as only 54% of children with HIV worldwide are on antiretroviral therapy [31].

The causes of death among children born to HIV-infected mothers to those born to HIV-negative mothers did not differ significantly. Though HIV-infected and exposed infants and children have impaired immune response, cause-specific mortality did not differ in these two groups in our study. Consistent with previous reports, respiratory diseases, malaria, and diarrheal illnesses were the leading causes of mortality among children in this study [32, 33]. Given that causes of death did not differ between HIV-exposed infants and children and those who were not infected, attention should be paid to child factors present at birth associated with mortality and not solely on specific disease processes to curb mortality rates among infants and children.

In our study, indicators of improved socioeconomic status were not associated with a lower risk of mortality among infants and children. This finding contrasts findings from previous reports from Tanzania, Uganda, and Nigeria [14, 34, 35]. Socioeconomic status is a complex and multifactorial structure and lacks a standardized definition used in research. Our findings may differ from prior studies in sub-Saharan Africa as other studies included populations up to age 5, differing access to medical care, sanitation, and nutrition. Further studies are merited to evaluate the effect of socioeconomic status on infant and child mortality in sub-Saharan Africa.

The presence of Apgar scores ≤7 at 5 min and birthweight of <2500 g were also associated with greater mortality among infants and children in our cohort. The association between Apgar score and mortality could partly be explained by the fact that at least two of the components of the Apgar score are dependent on respiratory function and the literature suggests that low Apgar score is significantly associated with morbidity [36–38]. The association of low birthweight with greater mortality risk in our study may be due to the relation of low birthweight and HIV-exposure and infection due to intrauterine growth restriction [39, 40]. However, on multivariable regression, low birthweight was an independent risk factor for mortality among infants and children.

## Limitations

The results of this secondary analysis should be interpreted with acknowledgement of their limitations. First, this cohort study was conducted prior to national guideline changes in Tanzania regarding the treatment of children with HIV. This may lead to reporting of higher mortality rates among HIV-infected infants and children than may be expected in the era of universal testing and treatment. Notwithstanding, the risk factors for mortality observed in this study may mirror those present in contemporary settings with low antiretroviral coverage. Second, our data come from two clinical trials with micronutrient supplementation and as such its results may not be generalizable to other populations, but neither of these trials reported a significant impact of these supplements on mortality. However, our study does provide insight into upstream factors associated with mortality that previous studies have not been designed to describe. Lastly, we did not have access to information about the initiation of antiretroviral therapy among infants and children born to mothers who were HIV-positive.

## Conclusions

HIV-infected infants and children had significantly higher mortality rates than those who were not infected in this large cohort study. Programs to address prevention of mother to child transmission and provision of care and treatment for HIV should be promoted to reduce mortality among infants and children. Moreover, infants and children who are HIV-exposed but uninfected carry a greater risk of mortality. Further studies are needed to assess interventions that may reduce mortality in this vulnerable population. Continued action in promoting high-quality maternal and newborn care should be implemented to improve postnatal outcomes.

## Data Availability

The datasets analyzed during this current study are available on reasonable request.

## Abbreviations

aHR: Adjusted hazards ratio
CI: Confidence interval
DNA: Deoxynucleic acid
ELISA: Enzyme-linked immunoassay
HIV: Human immunodeficiency virus
HR: Hazards ratio
MUAC: Mid-upper arm circumference
WHO: World Health Organisation
